# Production of neoagarooligosaccharides by probiotic yeast *Saccharomyces cerevisiae* var. *boulardii* engineered as a microbial cell factory

**DOI:** 10.1186/s12934-021-01644-w

**Published:** 2021-08-18

**Authors:** Yerin Jin, Sora Yu, Jing-Jing Liu, Eun Ju Yun, Jae Won Lee, Yong-Su Jin, Kyoung Heon Kim

**Affiliations:** 1 Department of Biotechnology, Graduate School, Korea University, Seoul, 02841 South Korea; 2 Carl R. Woese Institute for Genomic Biology, University of Illinois at Urbana-Champaign, Urbana, IL 61801 USA; 3 Department of Food Science and Human Nutrition, University of Illinois at Urbana-Champaign, Urbana, IL 61801 USA

**Keywords:** *Bp*GH16A, *Saccharomyces boulardii*, CRISPR-Cas9, Neoagarooligosaccharides, Probiotics, Prebiotics

## Abstract

**Background:**

*Saccharomyces cerevisiae* var. *boulardii* is a representative probiotic yeast that has been widely used in the food and pharmaceutical industries. However, *S. boulardii* has not been studied as a microbial cell factory for producing useful substances. Agarose, a major component of red macroalgae, can be depolymerized into neoagarooligosaccharides (NAOSs) by an endo-type β-agarase. NAOSs, including neoagarotetraose (NeoDP4), are known to be health-benefiting substances owing to their prebiotic effect. Thus, NAOS production in the gut is required. In this study, the probiotic yeast *S. boulardii* was engineered to produce NAOSs by expressing an endo-type β-agarase, *Bp*GH16A, derived from a human gut bacterium *Bacteroides plebeius.*

**Results:**

In total, four different signal peptides were compared in *S. boulardii* for protein (*Bp*GH16A) secretion for the first time. The SED1 signal peptide derived from *Saccharomyces cerevisiae* was selected as optimal for extracellular production of NeoDP4 from agarose. Expression of *Bp*GH16A was performed in two ways using the plasmid vector system and the clustered regularly interspaced short palindromic repeat (CRISPR)-Cas9 system. The production of NeoDP4 by engineered *S. boulardii* was verified and quantified. NeoDP4 was produced by *S. boulardii* engineered using the plasmid vector system and CRISPR-Cas9 at 1.86 and 0.80 g/L in a 72-h fermentation, respectively.

**Conclusions:**

This is the first report on NAOS production using the probiotic yeast *S. boulardii*. Our results suggest that *S. boulardii* can be considered a microbial cell factory to produce health-beneficial substances in the human gut.

**Graphical abstract:**

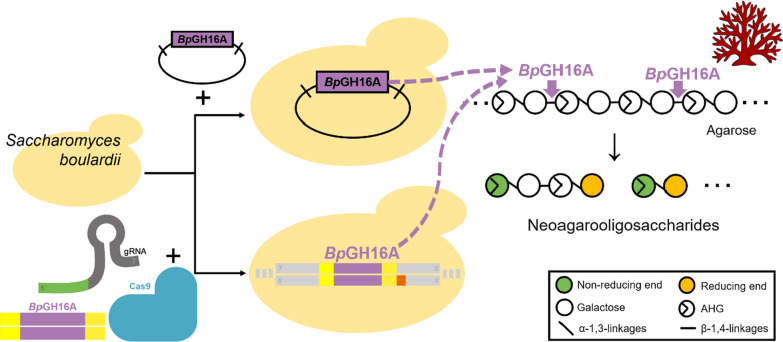

**Supplementary Information:**

The online version contains supplementary material available at 10.1186/s12934-021-01644-w.

## Background

The gut microbiota and intestinal immunity have been found to have a major impact on human health [1, 2]. Microbial cell factories, including *Saccharomyces cerevisiae*, have been approved for human use and have been developed to produce therapeutic proteins [3, 4]. Thus, if a microbial cell factory that can directly produce therapeutic proteins in the intestine is developed, it would be possible to study the effects of intestinal microbes on health more accurately, and the microbial cell factory could be used for the treatment of actual diseases. When designing microbial cell factories, one important consideration is the selection of an appropriate microbial host as an expression system [5], based on its safety and health benefits. Therefore, well-known probiotics, such as lactic acid bacteria and *Saccharomyces cerevisiae* var. *boulardii*, could be promising hosts as expression systems.

Lactic acid bacteria or intestinal bacteria can be used as hosts for microbial cell factories, but there may be some constraints. First, from a pharmacokinetic point of view, it may be necessary to control the inhabiting population and residence time in the gut [6]. In the case of *S. boulardii*, this yeast was washed out in the gut within 3–5 days after discontinuing oral administration [7, 8]. However, in the case of intestinal bacteria, it is not possible to control their inhabiting population and residence time in the gut. Second, post-translational modifications including glycosylation and phosphorylation of eukaryotic proteins, are crucial for the expression of their biological activity [9]. Therefore, yeast has been used as an eukaryotic host to produce many therapeutic proteins [9]. Taken together, the eukaryotic probiotic *S. boulardii* is considered a promising host to use as an intestinal microbial cell factory [10, 11].

*S. boulardii*, originally isolated from lychee and mangosteen, is a generally recognized as safe (GRAS) yeast [12, 13]. *S. boulardii* is known to survive in the human gastrointestinal tract owing to its high tolerance to low pH and high temperatures [13, 14]. Additionally, *S. boulardii* is the only probiotic yeast found to be effective in double-blind studies [14, 15]. Previously, the metabolic engineering of *S. boulardii* and its use as a potential oral vaccine delivery vehicle were studied in mouse models [16, 17]. However, to our knowledge, there have been no studies yet on the production of prebiotics which possess a beneficial effect on the health of a host by selective stimulation of the activity or growth of probiotic-like bacteria in the colon using an engineered *S. boulardii* [18]. In this study, we engineered the probiotic yeast *S. boulardii* to produce bioactive substances with prebiotic effects with the ultimate goal of developing a synbiotic system for humans.

When non-digestible diets reach the large intestine, they are utilized by the gut microbiota. Subsequently, non-digestible diets change the intestinal microflora and affect the overall health of the host [19, 20]. As a non-digestible diet, agarose, a polysaccharide obtained from red macroalgae, is commonly used as dietary fiber by East Asians [21]. Neoagarooligosaccharides (NAOSs) derived from agarose were found to have various physiological and biological activities, including anti-obesity, anti-diabetic, anti-inflammatory, anti-viral, and anti-tumor activities [22–27]. Moreover, in vivo experiments confirmed that NAOSs have a prebiotic effect [28]. In particular, neoagarotetraose (NeoDP4), which contains various bioactive properties, such as anti-inflammatory [29] and anti-oxidative activity [30], has been found to be a potential prebiotic for modulating intestinal microbiota, thereby promoting the health of the host. In addition, *Bifidobacterium*, which is considered as a beneficial probiotic microorganism having therapeutic benefits and is one of the most commonly used probiotics in humans [31], significantly increased in mice treated with antibiotics supplemented with NeoDP4 [29]. NeoDP4 can be produced by endo-type β-agarase from agarose. Recently, an endo-type β-agarase, *Bp*GH16A originating from human gut bacterium *Bacteroides plebeius*, has been reported [32, 33]. As *B. plebeius* was isolated from human gut microbes that can be considered relatively safe, *Bp*GH16A was chosen to be expressed in *S. boulardii* to enzymatically produce NAOSs, primarily NeoDP4, from agarose.

**Fig. 1:**
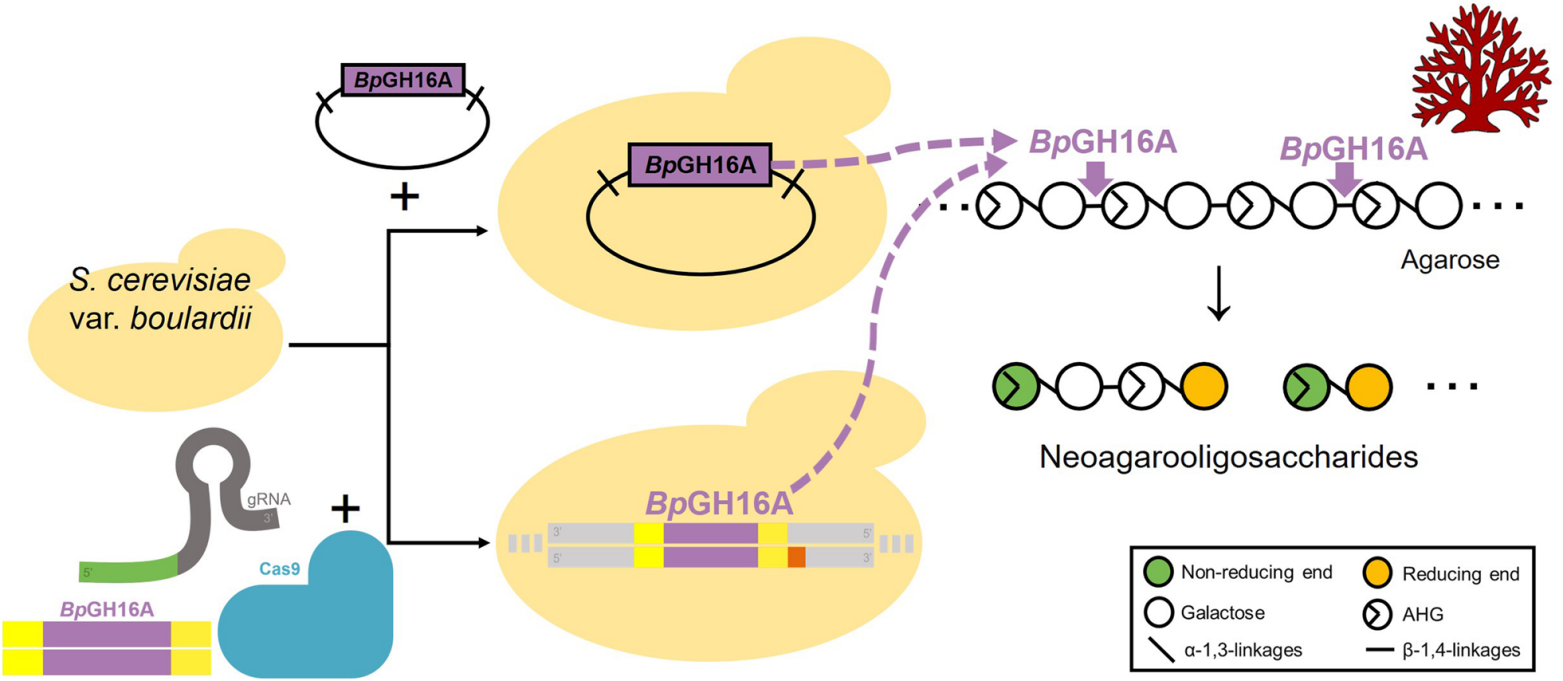
Schematic representation of NAOS production by engineered *S. boulardii*. *Bp*GH16A expression was performed using two different systems. The first was a vector system with an auxotrophic marker, and the second was the clustered regularly interspaced short palindromic repeat (CRISPR)-Cas9 system. NAOSs, neoagarooligosaccharides; *Bp*GH16A, endo-type β-agarase originating from human gut bacterium *Bacteroides plebeius*

In this study, we introduced and expressed the gene for *Bp*GH16A in the probiotic yeast *S. boulardii* using CRISPR-Cas9, and the production of prebiotic NeoDP4 from agarose by the engineered *S. boulardii* was verified and optimized (Fig. 1). To our knowledge, this is the first study to show that the probiotic *S. boulardii* can be used as a microbial cell factory for producing prebiotics.

## Methods

### Strains and media

*Escherichia coli* DH5α was used to construct the plasmids. *E. coli* strains were grown in Luria-Bertani medium (10 g/L tryptone, 5 g/L yeast extract, and 10 g/L sodium chloride) containing 100 µg/mL ampicillin (Sigma-Aldrich, St. Louis, MO, USA) at 37 °C and 200 rpm. *S. boulardii* ATCC MYA-796 was used as the parental strain for producing NeoDP4. Yeast strains were grown at 37 °C on yeast synthetic complete (YSC) medium, which contained a 6.7 g/L yeast nitrogen base without amino acids (Becton, Dickinson and Company, Franklin Lakes, NJ, USA), 20 g/L glucose, and 1.92 g/L yeast synthetic drop-out medium supplements without uracil (Sigma-Aldrich), or YSC medium, which contained a 6.7 g/L yeast nitrogen base (Becton, Dickinson and Company) and 20 g/L glucose. For CRISPR-Cas9-based genome editing experiments, 100 µg/mL nourseothricin (NAT; Jena Bioscience, Jena, Germany) and 500 µg/mL geneticin (AG Scientific, San Diego, CA, USA) were added to the medium if required for selection of the transformants.

### Plasmid and strain construction

To use the auxotrophic marker for transformant selection, a strain with inactivated *HIS3*, *TRP1*, and *URA3* was created first. Based on the SB-TU strain [17] in which *TRP1* and *URA3* were inactivated, *HIS3* was further inactivated. The gene fragment of *HIS3* [34] was amplified using the primer pair gHIS3_F and gHIS3_R (Table 1). The resulting polymerase chain reaction (PCR) product was digested with SacI and NotI and ligated to pRS42H, resulting in plasmid p42H_gHIS3, designed for simultaneously inactivating *HIS3* (Table 2). Repair DNA for *HIS3* disruption was amplified using PCR by using primers dDNA_HIS3_F and dDNA_HIS3_R (Table 1). The Cas9-NAT plasmid conferring NAT resistance was used to select the transformants using an antibiotic marker after transformation [34]. Yeast transformation into SB-TU was carried out using the polyethyleneglycol (PEG)-LiAc method, as described previously [35]. Finally, *S. boulardii* strain SB-HTU, in which *HIS3*, *TRP1*, and *URA3* were inactivated, was constructed (Table 3).

**Table 1:** Primers used in this study

Primer	Sequence (5′→3′, restriction sites are underlined)
gHIS3_F	TCCACCTAGCGGATGACTCT
gHIS3_R	TGCATTACCTTGTCATCTTC
dDNA_HIS3_F	GTAAAGCGTATTACAAATGAAACCAAGATTCAGATTGCGATCTCTTTAAAGGGTTAACCC
dDNA_HIS3_R	TTCTGGGAAGATCGAGTGCTCTATCGCTAGGGGTTAACCCTTTAAAGAGATCGCAATCTG
16A_W/OSP_F_SpeI	ATA ACT AGT GCA GAA AAT TTA AAT AAT AAA TCA TAC GAG TG
16A_W/OSP_R_XhoI	ATA CTC GAG TTC TTC TGG GAC CAG TGT ATA AAC
16A_CL_F_SpeI	ATA ACT AGT ATG AGG TCT TTG CTA ATC TTG GTG CTT TGC TTC CTG CCC CTG GCT GCT CTG GGG GCA GAA AAT TTA AAT AAT AAA TCA TAC
16A_CL_R_XhoI	ATA CTC GAG TTC TTC TGG GAC CAG T
αMF_F_SpeI	ATA ACT AGT ATG AGA TTT CCT TCA ATT TTT ACT G
αMF_R_16A	GCA GAA AAT TTA AAT AAT AAA GCT TCA GCC TCT CTT TTC T
16A_F_ αMF	GAG AAA AGA GAG GCT GAA GCT GCA GAA AAT TTA AAT AAT AAA TCA TAC G
16A_R_αMF_BamHI	ATA GGA TCC TTC TTC TGG GAC CAG TGT AT
STA1_F_EcoRI	ATA GAA TTC ATG GTA GGC CTC AAA AAT C
STA1_16A_ R	GCA GAA AAT TTA AAT AAT AAA TCA TAC GAG TTT TTT CTG TCG CTG GAG C
16A_STA1_ F	GGC TCC AGC GAC AGA AAA AAG CAG AAA ATT TAA ATA ATA AAT CAT ACG AG
16A_ R_XhoI	ATA CTC GAG TTC TTC TGG GAC CAG TGT AT
16A_SED1_F_SpeI	ATA ACT AGT ATG AAA TTA TCA ACT GTC CTA TTA TCT GCC GGT TTA GCC TCG ACT ACT TTG GCC CAA GCA GAA AAT TTA AAT AAT AAA TCA
16A_SED1_R_XhoI	ATA CTC GAG TTC TTC TGG GAC CAG
dDNA-CS5-F	AAA AGA GAA GAA AAA AGA GAA GAA ATG AAT TCT ATT ATG ATA GCG AAT GCA ATT AAC CCT CAC TAA AGG GA
dDNA-CS5-R	TGC TGG TTG CCT TAT TAA TTT ATA TGG AAG ACG AGA TAA TTC ATT AAT TAG TAA TAC GAC TCA CTA TAG GGC
dDNA-CS5 + 60_F	ATG GTA CAC GCT CTT GGC AAC ATT GAA ATT ACA GCT CTC ATA TAT AAA AAA TGG AAA GAA AAA AGA GAA GAA AAA AGA GAA GAA ATG AAT
dDNA-CS5 + 60_R	GGC ATA ACA ATA GCG CAC AGA TCC GCA GGT TTC GTA ATA CGC TTA ACA ATA GGC GTC TCC TGC TGG TTG CCT TAT TAA TTT ATA TGG AAG
gCS5_F	CTG GTA GTT GCA CAG AAA GAG TTT TAG AGC TAG AAA TAG CAA G
gCS5_R	TCT TTC TGT GCA ACT ACC AGC GAT CAT TTA TCT TTC ACT GCG
Conf-CS5-F	AAT GAA TTC TAT TAT GAT AGC GAA TGC
Conf-CS5-R	CAC AGG ATT TAC GAA GAC C

**Table 2:** Plasmids used in this study

Plasmid	Description	References
pRS42H	2µ origin	EUROSCARF
p42H_gHIS3	pRS42H carrying *HIS3* disruption gRNA cassette	This study
pRS426GPD	*URA3*, GPD promoter, CYC1 terminator, 2µ origin, and Amp	Mumberg et al. [45]
p426_Bp_W/OSP	pRS426GPD harboring *Bp*GH16A from *B. plebeius*, deletion signal peptide	This study
p426_Bp_CL	pRS426GPD harboring *Bp*GH16A and chicken lysozyme signal peptide	This study
p426_Bp_αMF	pRS426GPD harboring *Bp*GH16A and α-mating factor signal peptide	This study
p426_Bp_STA1	pRS426GPD harboring *Bp*GH16A and *STA1* signal peptide	This study
p426_Bp_SED1	pRS426GPD harboring *Bp*GH16A and *SED1* signal peptide	This study
Cas9-NAT	p414-TEF1p-Cas9-CYC1t-NAT1	Zhang et al. [34]
pRS42K	2µ origin, KanMX	Taxis and Knop [46]
p42K_CS5	pRS42K, gRNA cassette targeting the intergenic site on Chr XV	This study
16 A-D-CS5	*Bp*GH16A, *SED1* signal peptide, donor DNA for CS5 site integration	This study

**Table 3:** Strains used in this study

Strain	Description	References
*S. boulardii*	ATCC MYA-796	ATCC
SB-TU	*S. boulardii*; *TRP1* and *URA3* disruption	Liu et al. [17]
SB-HTU	*S. boulardii*; *HIS3*, *TRP1*, and *URA3* disruption	This study
SB-HTU_16A_E	SB-HTU; pRS426GPD	This study
SB-HTU_16A_W	SB-HTU; *Bp*GH16A, deletion signal peptide, pRS426GPD	This study
SB-HTU_16A_C	SB-HTU; *Bp*GH16A, chicken lysozyme signal peptide, pRS426GPD	This study
SB-HTU_16A_A	SB-HTU; *Bp*GH16A, α-mating factor signal peptide, pRS426GPD	This study
SB-HTU_16A_S	SB-HTU; *Bp*GH16A, *STA1* signal peptide, pRS426GPD	This study
SB-HTU_16A_D	SB-HTU; *Bp*GH16A, *SED1* signal peptide, pRS426GPD	This study
SB_16A_D	*S. boulardii*; *Bp*GH16A, *SED1* signal peptide	This study

Plasmids that were used to screen an optimal signal peptide for secretion of *Bp*GH16A were constructed as follows. The gene BACPLE_01670, encoding *Bp*GH16A, was cloned into the pRS426GPD plasmid. The *Bp*GH16A gene fragment was amplified from *B. plebeius* DSM 17135 (DSMZ, Braunschweig, Germany) genomic DNA using PCR with different primer pairs depending on the type of signal peptide (Table 1). The predicted signal peptide sequences at the N-terminus of *Bp*GH16A were removed for signal peptide screening. In total, four different signal peptides, namely chicken lysozyme signal peptide (CL), α-mating factor signal peptide (α-MF) from *S. cerevisiae*, Sta1 signal peptide (STA1) from *Saccharomyces diastaticus*, and Sed1 signal peptide (SED1) from *S. cerevisiae*, were used [17, 36, 37]. Additionally, for construction of the control strain without any signal peptide, PCR was performed using the primer pairs 16A_W/OSP_F_SpeI and 16A_W/OSP_R_XhoI (Table 1). The PCR products were double-digested by restriction enzymes determined during primer design and ligated with plasmid pRS426GPD digested using the same restriction enzymes, using T4 DNA ligase (New England Biolabs, Ipswich, MA, USA). The resulting plasmids were designated as p426_Bp_W/OSP, p426_Bp_CL, p426_Bp_αMF, p426_Bp_STA1, and p426_Bp_SED1 (Table 2). Yeast transformation into SB-HTU was performed using the PEG-LiAc method. Finally, the experimental strains SB-HTU_16A_C, SB-HTU_16A_A, SB-HTU_16A_S, and SB-HTU_16A_D were prepared for signal peptide screening (Table 3). As control strains, SB-HTU_E harboring neither *Bp*GH16A nor signal peptide, and only pRS426GPD vector, and SB-HTU_W harboring the *Bp*GH16A gene but no signal peptide were prepared.

To integrate the *Bp*GH16A gene into the genome of *S. boulardii* for the stable expression of *Bp*GH16A, a guide RNA plasmid p42K_CS5 was constructed (Table 2). The plasmid was generated using reverse PCR of the pRS42K plasmid containing a guide RNA sequence using the primer pairs gRNA_CS5_F and gRNA_CS5_R (Table 1). The 20-bp targeting sequence of the guide RNA binds to the front of the PAM sequence (NGG) in the empty locus on chromosome XV (CS5). The *Bp*GH16A and *SED1* signal peptide were incorporated into this locus via homologous recombination without affecting the function of other genes. For homologous recombination, the plasmid p426_Bp_SED1 was amplified using the primer pairs dDNA-CS5-F and dDNA-CS5-R as donor DNA for genome integration using CRISPR-Cas9 (Table 1). To overcome the inefficiencies associated with genome integration, the PCR product constructed using the primer pairs dDNA-CS5-F and dDNA-CS5-R was amplified using PCR by using primer pairs CS5 + 60_F and CS5 + 60_R (Table 1). The homologous region was found to be approximately 120 bp. During yeast transformation, 1 µg of Cas9-NAT plasmid, 20 µg of 16 A-D-CS5 with donor DNA, and 2 µg of p42K_CS5 with guide RNA were added to *S. boulardii* and transformed. Genomic integration was verified with yeast colony PCR by using the primer pairs Conf-CS5-F and Conf-CS5-R (Table 1).

### Fermentation experiments

To produce NAOSs in the engineered *S. boulardii*, fermentation was performed with 2.5 g/L agarose at 37 °C and 200 rpm in 125-mL flasks for 72 h. During fermentation, agarose, low gelling temperature (Sigma-Aldrich) was used to prevent congealing of agarose. First, strains SB-HTU_16A_C, SB-HTU_16A_A, SB-HTU_16A_S, and SB-HTU_16A_D were grown in YSC medium at 37 °C and 200 rpm. Pre-cultured cells were centrifuged at 10,170 × *g* for 10 min and washed twice using sterilized distilled water. The harvested cells were inoculated into 20 mL of YSC medium containing 20 g/L glucose and 2.5 g/L agarose in 50 mM potassium hydrogen phthalate (KHP) buffer (pH 5.5). The initial cell density was adjusted to an optical density at 600 nm (OD_600_) of 1.0. As a control, strains SB-HTU_16A_E and SB-HTU_16A_W were fermented under the same conditions.

To examine NAOSs production by *S. boulardii* SB_16A_D engineered by CRISPR-Cas9, fermentation was performed in YSC medium. Pre-cultured cells in YSC medium were inoculated in 20 mL of YSC medium containing 20 g/L glucose and 2.5 g/L agarose in 50 mM KHP buffer (pH 5.5). The initial cell density was adjusted to OD_600_ = 1.0. Wild-type *S. boulardii* ATCC MYA-796 was used as the control strain. Fermentation experiments were performed in triplicates.

### Analyses of cell growth and NAOS production using high-performance liquid chromatography

Cell growth was monitored by measuring OD_600_ using a UV-visible spectrophotometer (Bio-Rad, Hercules, CA, USA). To analyze and quantify the reaction products of agarose by the engineered *S. boulardii*, including NeoDP4, glucose, acetic acid, and ethanol, high-performance liquid chromatography (HPLC) analysis was performed. The HPLC system (Agilent Technologies, Santa Clara, CA, USA) was equipped with a refractive index (RI) detector (Agilent Technologies) using an Aminex HPX-87H column (Bio-Rad). The column and RI detector temperatures were set to 65 and 55 °C, respectively, and the column was eluted with 0.005 M H_2_SO_4_ at a flow rate of 0.5 mL/min.

### Identification of NAOSs using thin-layer chromatography

To identify the hydrolyzed products of agarose during fermentation, thin-layer chromatography (TLC) analysis was performed. During fermentation, 1 mL of cell culture containing the fermentation products was obtained for each time point (0, 12, 24, 36, 48, and 72 h). For accurate measurements, the obtained cell culture was boiled to terminate the possible enzymatic reaction. After centrifugation at 16,609×*g* for 15 min at 4 °C, a 1-µL aliquot from each supernatant was loaded onto silica gel 60 plates (Merck, Damstadt, Germany). After drying the TLC plates, they were visualized with 10% (v/v) H_2_SO_4_ in ethanol and 0.2% (w/v) naphthoresorcinol in ethanol, as previously described [38].

## Results

### NeoDP4 production by engineered yeast

To produce NeoDP4 by engineered yeast, β-agarase secretion from yeast was necessary for agarose degradation. Therefore, the expression and secretion of an endo-type β-agarase, *Bp*GH16A, by yeast *S. boulardii*, was tested first. To confirm that *Bp*GH16A is functionally expressed and secreted by *S. boulardii*, SB-HTU_16A_C containing CL, which has been previously proven to work in *S. boulardii*, was used [17].

**Fig. 2:**
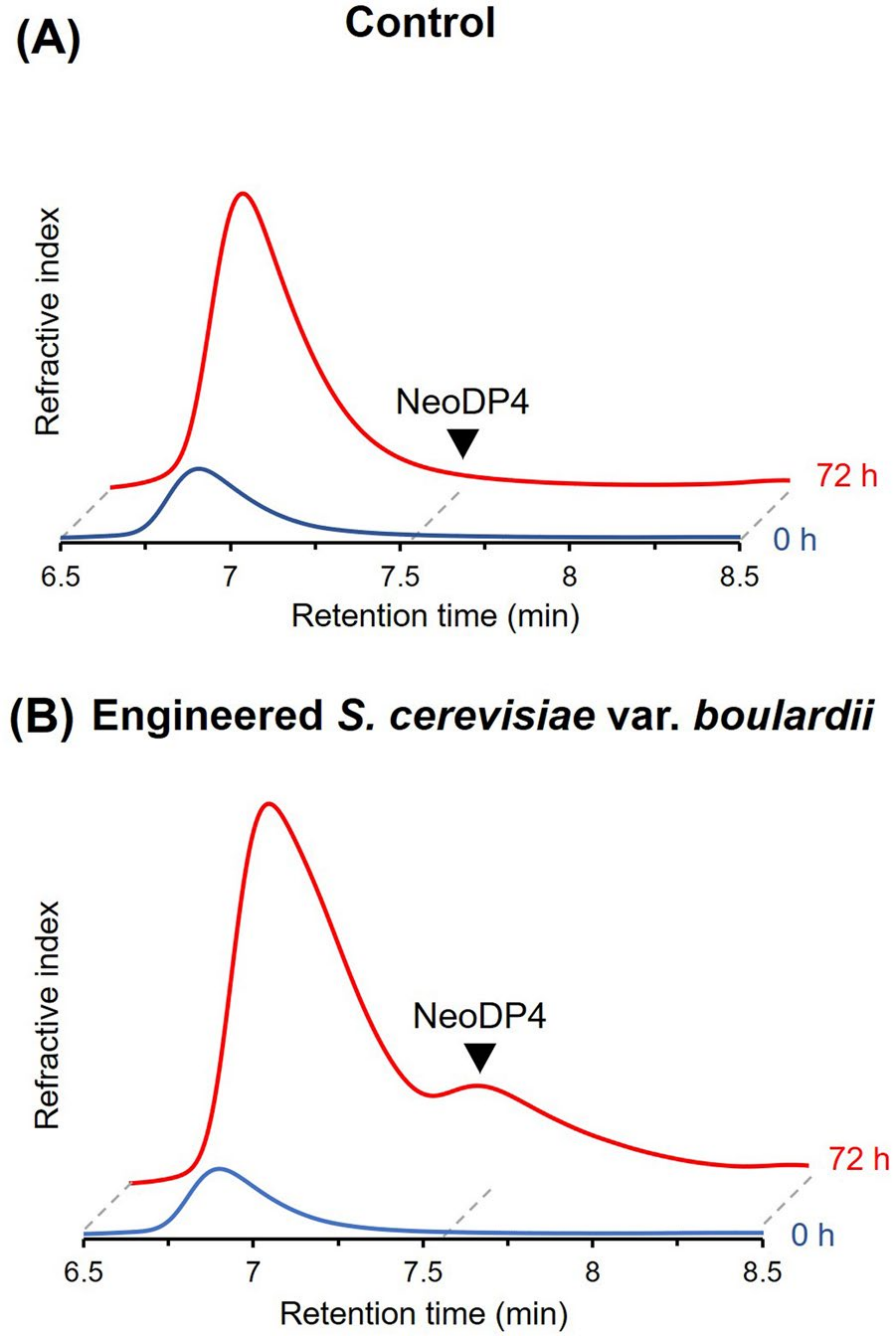
Identification of NeoDP4 produced via fermentation by the engineered yeast *S. boulardii* SB-HTU_16A_C strain using HPLC. HPLC chromatograms of (**A**) control and (**B**) engineered *S. boulardii* SB-HTU_16A_C strains at 72 h of fermentation. The peak of NeoDP4 showed a retention time approximately at 7.6 min. Control, SB-HTU_16A_E harboring the empty plasmid pRS426GPD; the engineered *S. boulardii*, SB-HTU_16A_C harboring p426_Bp_CL; NeoDP4, neoagarotetraose

In the HPLC analysis of SB-HTU_16A_C fermentation products, a significant peak at an approximate retention time of 7.6 min, corresponding to NeoDP4, was detected in the sample (Fig. 2). In contrast, no peak was detected in the sample from the control strain, SB-HTU_16A_E, harboring the empty vector. Thus, these results showed that *Bp*GH16A was functionally expressed, secreted from *S. boulardii*, and produced NeoDP4 by hydrolyzing agarose.

### **Screening of the optimal signal peptide for NeoDP4 production by*S. boulardii***

**Fig. 3:**
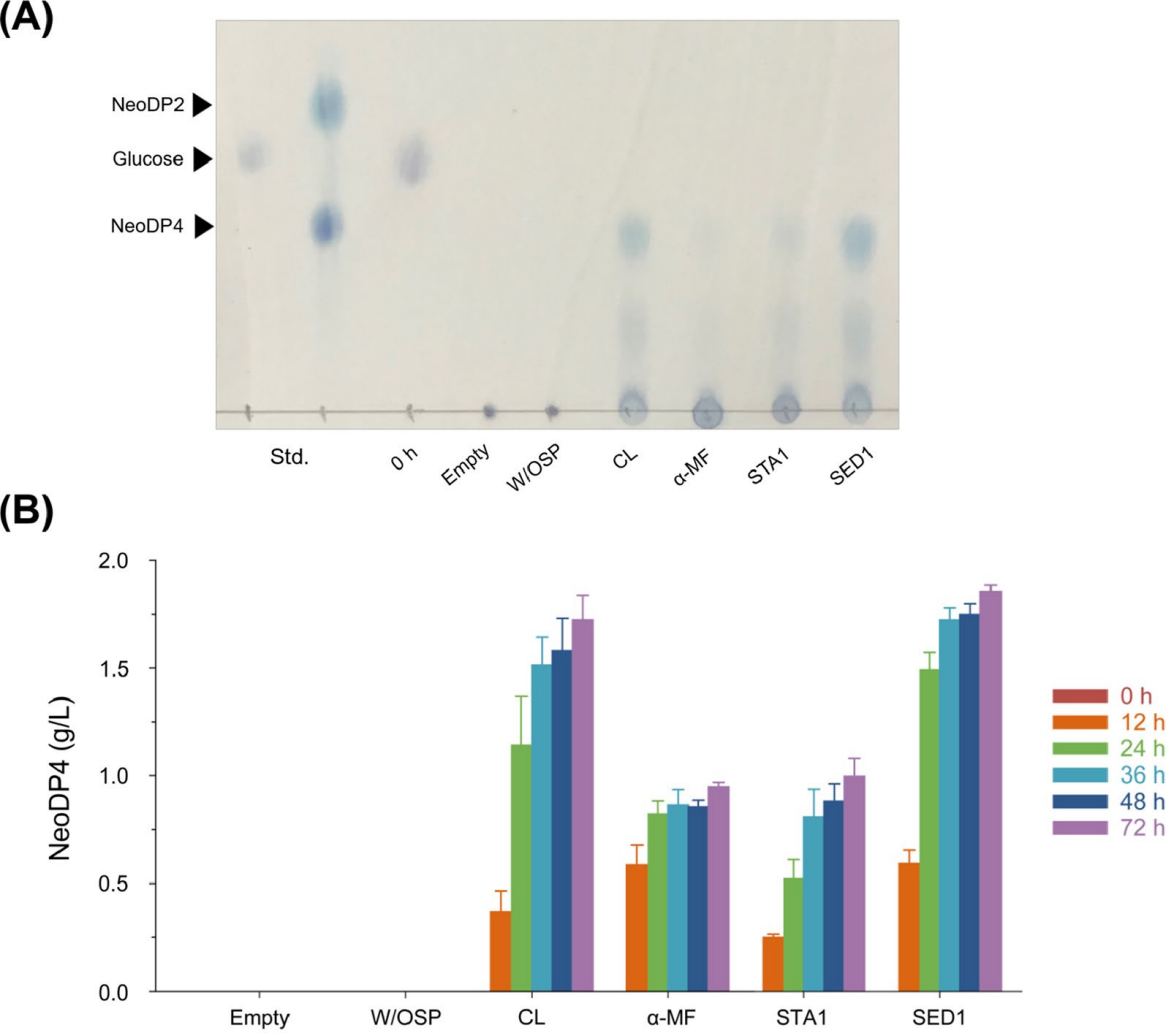
Signal peptide screening for the production of NeoDP4 in *S. boulardii*. **A** TLC analysis of the fermentation products by engineered yeast strains. **B** Comparison of the extracellular NeoDP4 concentration depending on the origin of the signal peptide sequences. Empty, SB-HTU_16A_E harboring pRS426GPD; W/OSP, SB-HTU_16A_W harboring p426_Bp_W/OSP; CL, SB-HTU_16A_C harboring p426_Bp_CL; α-MF, SB-HTU_16A_A harboring p426_Bp_αMF; STA1, SB-HTU_16A_S harboring p426_Bp_STA1; SED1, SB-HTU_16A_D harboring p426_Bp_SED1. Engineered strains were cultured with 20 g/L glucose and 2.5 g/L agarose in 50 mM KHP buffer (pH 5.5) at 37 °C and 200 rpm for 72 h. Results are presented as the mean values and standard deviations of data from three independent biological replicates. NeoDP4, neoagarotetraose; NeoDP2, neoagarobiose

As the enzyme *Bp*GH16A was confirmed to be functionally expressed and secreted by *S. boulardii*, the next step was to identify the optimal signal peptide to further increase NeoDP4 production. Four different signal peptides, CL, α-MF, STA1, and SED1, were tested. Each signal peptide was individually pre-fixed to the *Bp*GH16A sequences and introduced into SB-HTU to identify the signal peptide that produces more NeoDP4. Production of NeoDP4 by the engineered yeasts was verified using TLC analysis at 72 h (Fig. 3A); NeoDP4 was strongly produced by the strains containing CL and SED1. The NeoDP4 spot was weakly detected by the strain with α-MF. To compare the amount of NeoDP4 produced by each engineered yeast more accurately, HPLC analysis was performed (Fig. 3B). NeoDP4 was found to have gradually increased during the 72-h fermentation by each strain, and the highest amount of NeoDP4 was produced by the strain containing SED1. The amount of NeoDP4 produced after 72 h of fermentation was 1.73, 0.95, 0.99, and 1.86 g/L when signal peptides, namely CL, α-MF, STA1, and SED1, respectively, were used. The production of NeoDP4 by the strain containing SED1 was 1.08, 1.96, and 1.88 times higher than that by the strains containing CL, α-MF, and STA1, respectively. Thus, SED1 from *S. cerevisiae*, which showed the highest production of NeoDP4, was selected for further NeoDP4 production.

Meanwhile, NeoDP4 was not produced in any control group, whereas it was produced in all groups in which the signal peptide was present (Fig. 3). In particular, SB-HTU_16A_W, which was used as a negative control, was constructed to confirm that the extracellular activity of *Bp*GH16A was not derived from cell lysis but from secretion due to the heterologously expressed signal peptides. As NeoDP4 was not detected in the culture broth of SB-HTU_16A_W, the degradation of agarose into NeoDP4 was confirmed to be caused by the secreted *Bp*GH16A.

### Fermentation for the production of NeoDP4 by the engineered yeast

**Fig. 4:**
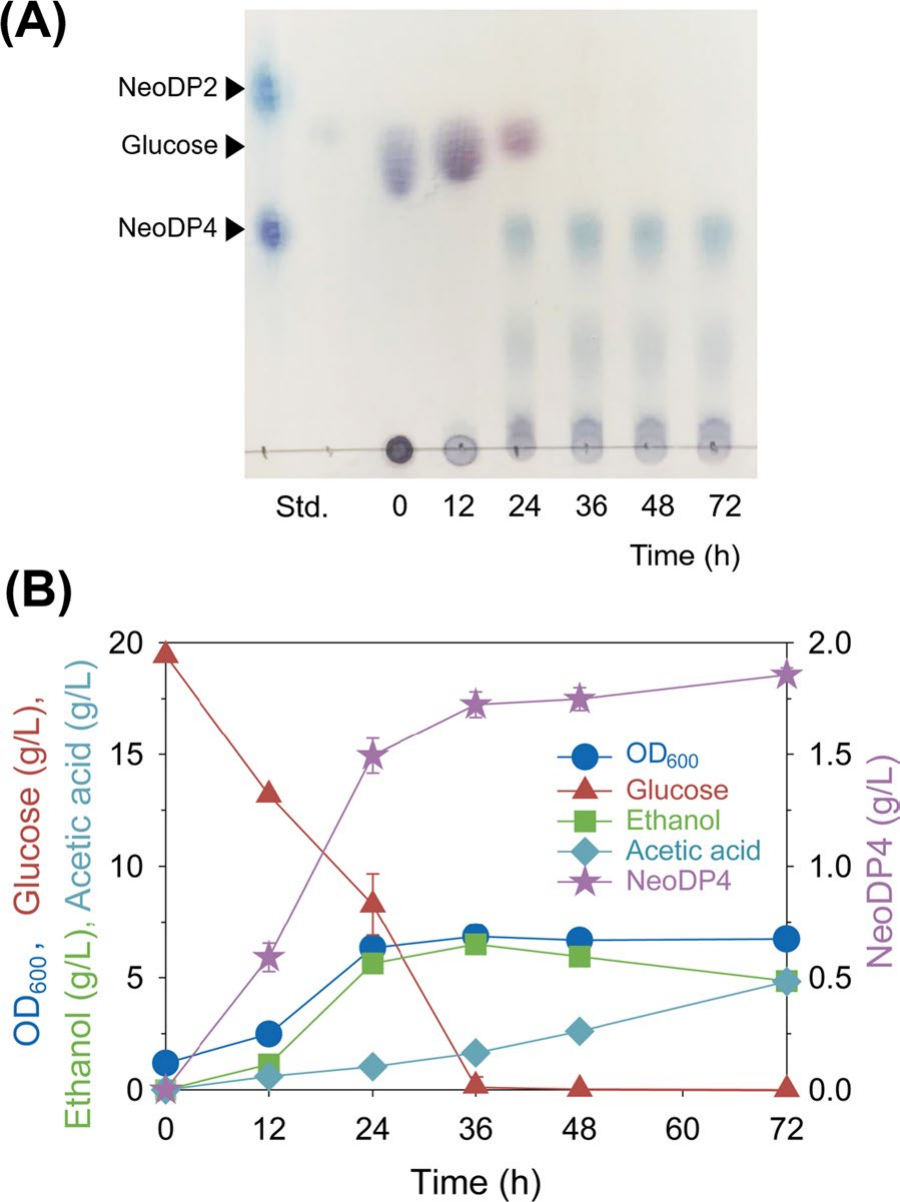
Fermentation profiles of engineered *S. boulardii* SB-HTU_16A_D harboring p426_Bp_SED1. **A** Time course analysis of the fermentation products of SB-HTU_16A_D using TLC. **B** Fermentation profiles of engineered *S. boulardii* SB-HTU_16A_D in YSC medium containing 20 g/L glucose and 2.5 g/L agarose in 50 mM KHP buffer (pH 5.5) at 37 °C and 200 rpm for 72 h. During fermentation, the cell density (OD_600_) and the concentration values of glucose, ethanol, acetic acid, and NeoDP4 were monitored using HPLC. All data points are the means of experimental data from triplicate fermentations. SB-HTU_16A_D harboring p426_Bp_SED1. NeoDP4, neoagarotetraose; NeoDP2, neoagarobiose; YSC, yeast synthetic complete

Based on the signal peptide screening results, the strain SB-HTU_16A_D containing the SED1 signal peptide was fermented in YSC medium without uracil containing 2.5 g/L agarose for 72 h. The fermentation products were analyzed using TLC and HPLC. NeoDP4 production was confirmed using TLC analysis (Fig. 4A). The initially added glucose was confirmed to be depleted after 36 h. Based on HPLC analysis of the fermentation products at each time point, 1.86 g/L NeoDP4 was obtained after 72 h of fermentation (Fig. 4B). Cell growth entered the stationary phase from 24 h onwards and reached an OD_600_ = 6.7. Both ethanol and acetic acid accumulated up to a concentration of 4.8 g/L. In conclusion, NeoDP4 was produced as the target major product by the engineered yeast SB-HTU_16A_D.

### Strain construction using CRISPR-Cas9 and fermentation

For stable expression, *Bp*GH16A and SED1 were introduced into the genome of *S. boulardii*, in this study. *Bp*GH16A gene knock-in was performed via homologous recombination using CRISPR-Cas9 (Additional file 1: Fig. S1A). After yeast transformation, the gene encoding *Bp*GH16A was confirmed to have entered the genome successfully, using yeast colony PCR. Primer pairs Conf-CS5-F and Conf-CS5-R were designed and used so that the size was 2.4-kb when the gene entered and 0.5-kb when the gene was not entered (Table 2). The successful integration of *Bp*GH16A and SED1 into *S. boulardii* was confirmed with yeast colony PCR, based on the formation of a 2.4-kb single band in 3 lanes, namely, 4, 7, and 8 out of total 9 lanes (Additional file 1: Fig. S1B).

**Fig. 5:**
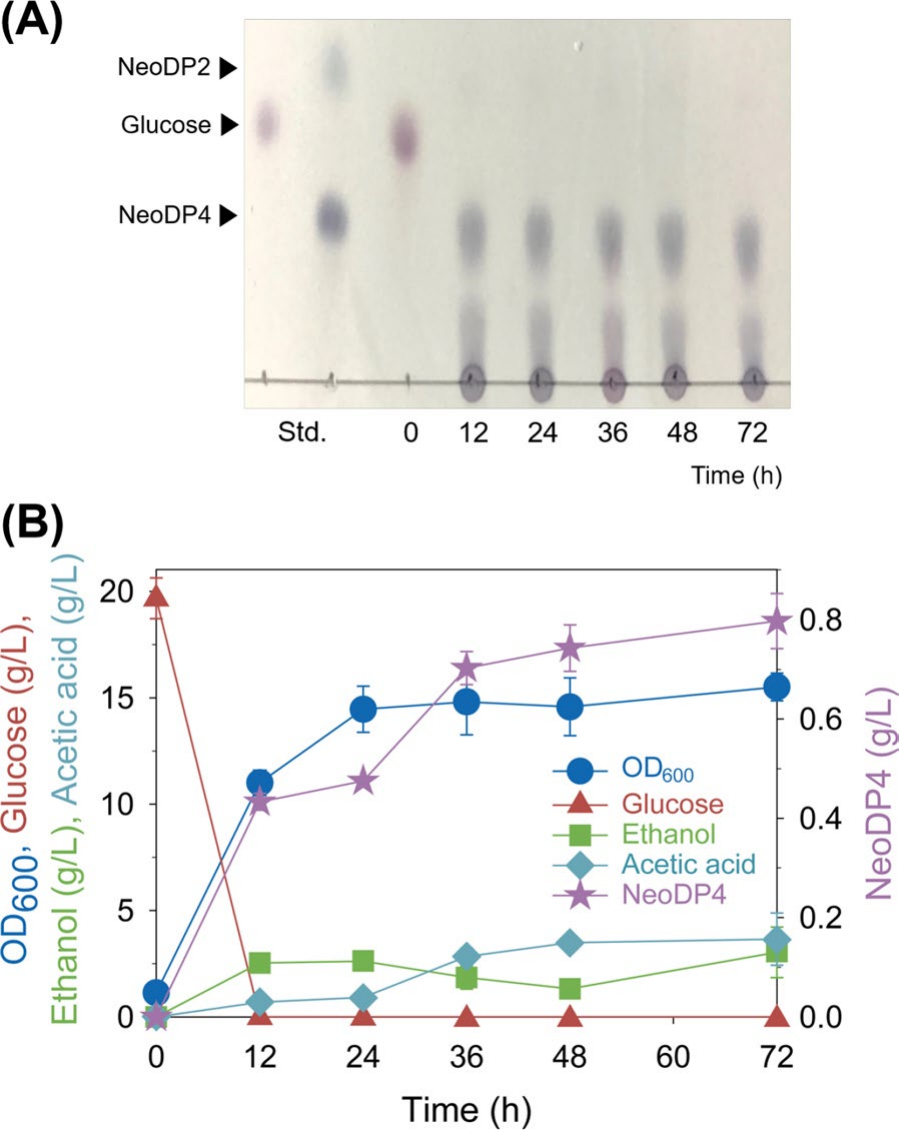
Fermentation profiles of *S. boulardii* SB_16A_D engineered using CRISPR-Cas9. **A** Time course analysis of the fermentation products of SB_16A_D using TLC. **B** Fermentation profiles of engineered *S. boulardii* SB_16A_D in YSC medium containing 2.5 g/L agarose in 50 mM KHP buffer (pH 5.5) at 37 °C and 200 rpm for 72 h. During fermentation, the cell density (OD_600_) and the concentration values of glucose, acetic acid, ethanol, and NeoDP4 were monitored. All data points are the means of experimental data from fermentations in triplicate. SB_16A_D, *S. boulardii* expressing *Bp*GH16A with SED1 signal peptide sequences using CRISPR-Cas9; NeoDP4, neoagarotetraose; NeoDP2, neoagarobiose; Std., standards (size markers)

Finally, strain SB_16A_D containing *Bp*GH16A and SED1 in the *S. boulardii* genome was constructed using CRISPR-Cas9, and flask fermentation proceeded in YSC medium containing 2.5 g/L agarose, for 72 h. NeoDP4 production was confirmed using TLC analysis (Fig. 5A), suggesting that *Bp*GH16A was secreted from strain SB_16A_D. For more accurate fermentation products analysis, HPLC analysis and growth measurement were performed at each time point as well (Fig. 5B). Glucose was depleted before 12 h of fermentation had passed, and the strain grew to an OD_600_ = 15.51 at 72 h. After fermentation, 0.80 g/L NeoDP4 was produced, as well as 3.03 g/L ethanol and 3.65 g/L acetic acid.

## Discussion

*S. boulardii* has been widely used as a probiotic because it can compete with diarrhea-causing pathogens in the human gut [39]. Additionally, *S. boulardii* may be used as a host for a microbial cell factory that produces useful proteins in the gut [11]. *S. boulardii* has been metabolically engineered; however, it has not yet been engineered to produce prebiotics [17]. In this study, we introduced an endo-type β-agarase, *Bp*GH16A, into *S. boulardii* to produce potential prebiotics, NAOSs from red macroalgal agarose (Fig. 1). The production of NeoDP4, a NAOS, was verified by engineered *S. boulardii*.

NAOSs, which can be produced by hydrolyzing agarose extracted from red macroalgae by endo-type β-agarase, have been reported to possess various health benefits [22–27]. NeoDP4, which is a representative NAOS, has been reported to have various biological properties [40, 41]. For example, the anti-fatigue effects of NeoDP4 via short-chain fatty acid production and regulation of the microbial composition have been demonstrated in mice [29]. Moreover, a significant increase in *Lactobacillus* and *Bifidobacterium* was observed by supplementing mice with NeoDP4, implying that NeoDP4 is a potential prebiotic [29]. NeoDP4 is also known to alleviate the inflammatory response by inhibiting the MAPK and NF-κB signaling pathways [26]. Therefore, the requirement for production of NAOSs, especially NeoDP4, is increasing rapidly [42].

One of the goals of this study was to effectively secrete *Bp*GH16A to hydrolyze agarose into NeoDP4 by expressing a signal peptide in engineered yeast. Previous studies have used CL or α-MF signal peptides to secrete proteins from *S. boulardii* [11, 16, 17]. Based on additional screening using STA1 and SED1 used in other yeast strains [36, 37], all of them (CL, α-MF, STA1, and SED1) were confirmed to secrete *Bp*GH16A in *S. boulardii* (Fig. 3). Through signal peptide screening, SED1 was shown to have the highest efficiency in producing NeoDP4 via secretion of *Bp*GH16A. Because of the lack of other reports on signal peptide screening in *S. boulardii* so far, this study could contribute to studies on the production and secretion of other proteins by *S. boulardii*.

Genomic integration can avoid problems that may arise in a complex intestinal environment when using plasmids. These problems include plasmid instability in the absence of selective pressure, potential diffusion to other microorganisms, and an increased metabolic burden associated with the maintenance of multicopy plasmids [43]. Therefore, we attempted to integrate BACPLE_01670 coding for *Bp*GH16A into the genome of *S. boulardii* using the CRISPR-Cas9 system, which is a sophisticated and advanced genomic engineering tool (Additional file 1: Fig. S1A) [44], and succeeded in constructing an *S. boulardii* strain that secretes *Bp*GH16A, namely, SB_16A_D. SB_16A_D strain produced 0.80 g/L NeoDP4 (Fig. 5) at 72 h. Compared to that when using a plasmid vector system with an auxotrophic marker, the final OD_600_ after 72 h of fermentation of the strain constructed using the CRISPR-Cas9 system was 2.3 times higher, but the NeoDP4 production was lower. This difference was presumed to be due to the relatively strong constitutive promoter and the high copy number of the pRS426GPD plasmid [45]. Nevertheless, the successful protein secretion from *S. boulardii* using genomic integration showed that *S. boulardii* could be used as a microbial cell factory for producing useful proteins and their products in the human gut.

## Conclusions

We have, for the first time, demonstrated that NAOSs can be produced by the probiotic yeast *S. boulardii*. Our signal peptide screening results provide more options available in *S. boulardii*. We also succeeded in producing health beneficial substances using probiotic yeast harboring *Bp*GH16A, an endo-type β-agarase, originating from the human gut bacteria *B. plebeius*. Our results suggest that synbiotics can be achieved by engineered probiotic yeast that produce prebiotics in the human gut.

## Supplementary Information

**Additional file 1: Fig. S1.** Engineering of *S. boulardii* for NAOSs production using CRISPR-Cas9 system. (A) Diagram for the construction of engineered *S. boulardii* expressing *Bp*GH16A using the CRISPR-Cas9 system. (B) Yeast colony PCR for confirmation of the genomic integration of each mutant.

## Data Availability

The datasets used and/or analyzed during the current study are available from the corresponding authors upon reasonable request.

## Abbreviations

α-MF: α-Mating factor signal peptide
CL: Chicken lysozyme signal peptide
CRISPR: Clustered regularly interspaced short palindromic repeat
GH16: Glycoside hydrolase 16
GRAS: Generally regarded as safe
HPLC: High-performance liquid chromatography
KHP: Potassium hydrogen phthalate
NAOSs: Neoagarooligosaccharides
NAT: Nourseothricin
NeoDP4: Neoagarotetraose
OD_600_: Optical density at 600 nm
PCR: Polymerase chain reaction
PEG: Polyethylene glycol
RI: Refractive index
SED1: SED1 Signal peptide
STA1: STA1 Signal peptide
TLC: Thin-layer chromatography
YSC: Yeast synthetic complete
