# Supplementary material for: Production of neoagarooligosaccharides by probiotic yeast Saccharomyces cerevisiae var. boulardii engineered as a microbial cell factory
Source: Microb Cell Fact. 2021 Aug 18;20:160. doi: 10.1186/s12934-021-01644-w (PMC8371797; doi:10.1186/s12934-021-01644-w)
Supplement: Supplementary file 1 — Additional file 1: Fig. S1. Engineering of S. boulardii for NAOSs production using CRISPR-Cas9 system. (A) Diagram for the construction of engineered S. boulardii expressing BpGH16A using the CRISPR-Cas9 system. (B) Yeast colony PCR for confirmation of the genomic integration of each mutant. [file 12934_2021_1644_MOESM1_ESM.docx]

Additional file 1

**Production of neoagarooligosaccharides by probiotic yeast *Saccharomyces cerevisiae* var. *boulardii* engineered as a microbial cell factory**

Yerin Jin^1^†, Sora Yu^1†^, Jing-Jing Liu^2^, Eun Ju Yun^1^, Jae Won Lee^2^, Youg-Su Jin^2,3^*, and Kyoung Heon Kim^1*^

^1^Department of Biotechnology, Graduate School, Korea University, Seoul 02841, South Korea

^2^Carl R. Woese Institute for Genomic Biology, University of Illinois at Urbana-Champaign, Urbana, IL 61801, USA

^3^Department of Food Science and Human Nutrition, University of Illinois at Urbana-Champaign, Urbana, IL, 61801, USA

*Correspondence: [khekim@korea.ac.kr](mailto:khekim@korea.ac.kr) and [ysjin@illinois.edu](mailto:ysjin@illinois.edu)

†These authors contributed equally to this work.


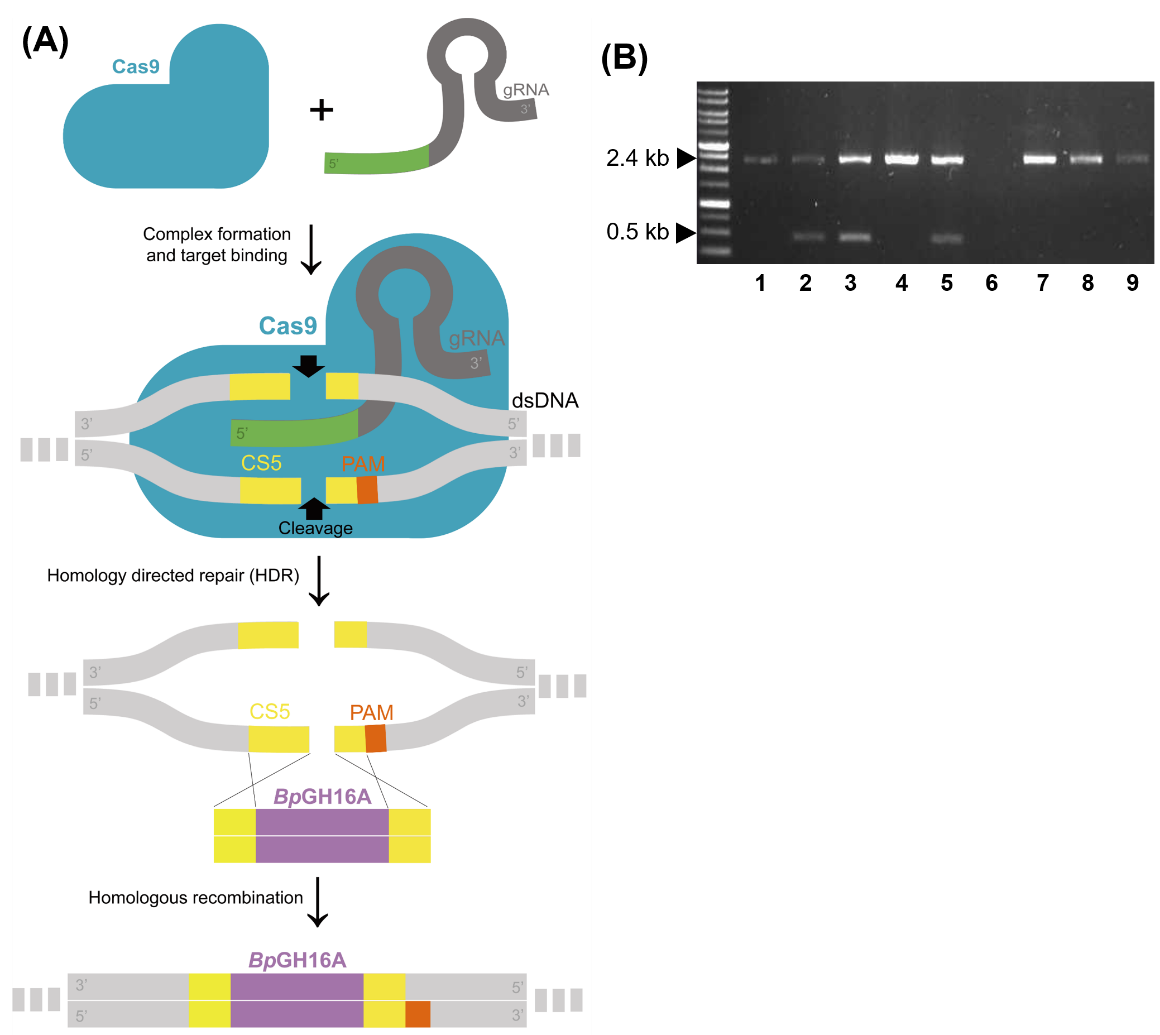


**Fig. S1** Engineering of *S. boulardii* for NAOSs production using CRISPR-Cas9 system. (A) Diagram for the construction of engineered *S. boulardii* expressing *Bp*GH16A using the CRISPR-Cas9 system. (B) Yeast colony PCR for confirmation of the genomic integration of each mutant.
